# Interactions between Diet and Exposure to Secondhand Smoke on the Prevalence of Childhood Obesity: Results from NHANES, 2007–2010

**DOI:** 10.1289/ehp.1510138

**Published:** 2015-12-29

**Authors:** Brianna F. Moore, Maggie L. Clark, Annette Bachand, Stephen J. Reynolds, Tracy L. Nelson, Jennifer L. Peel

**Affiliations:** 1 Department of Environmental and Radiological Health Sciences, and; 2 Department of Health and Exercise Science, Colorado State University, Fort Collins, Colorado, USA

## Abstract

**Background:**

Exposure to secondhand smoke (SHS) may increase risk for obesity, but few studies have investigated the joint effects of exposure to SHS and diet.

**Objectives:**

We examined the interaction of exposure to SHS and diet on the prevalence of obesity among 6- to 19-year-olds who participated in the 2007–2010 National Health and Nutrition Examination Survey.

**Methods:**

We characterized exposure using a novel biomarker [4-(methylnitrosamino)-1-(3-pyridyl)-1-butanol (NNAL)], an established biomarker (cotinine), and self-report. Multinomial logistic regression models examined the association of SHS exposure on the prevalence of overweight and obesity as separate outcomes (compared with normal/underweight). Interaction by diet was assessed by introducing interaction terms (with SHS) of the individual nutrients [dietary fiber, eicosapentaenoic acid (EPA), docosahexaenoic acid (DHA), vitamin C, and vitamin E] into separate models.

**Results:**

Approximately half of the children had NNAL and cotinine levels above the limit of detection, indicating exposure to SHS. Interaction results suggest that the prevalence of obesity among children with both high exposure to SHS and low levels of certain nutrients (dietary fiber, DHA, or EPA) is greater than would be expected due to the effects of the individual exposures alone. Little or no evidence suggesting more or less than additive or multiplicative interaction was observed for vitamin C or vitamin E. The association between SHS and obesity did not appear to be modified by dietary vitamin C or vitamin E.

**Conclusions:**

Childhood obesity prevention strategies aimed at reducing SHS exposures and improving diets may exceed the expected benefits based on targeting either risk factor alone.

**Citation:**

Moore BF, Clark ML, Bachand A, Reynolds SJ, Nelson TL, Peel JL. 2016. Interactions between diet and exposure to secondhand smoke on the prevalence of childhood obesity: results from NHANES, 2007–2010. Environ Health Perspect 124:1316–1322; http://dx.doi.org/10.1289/ehp.1510138

## Introduction

Obesity and obesity-related morbidity are global crises that affect all age groups (Karnik and Kanekar 2012), especially children (Wang and Lobstein 2006). Although the prevalence of obesity may be stabilizing in recent years (Skinner and Skelton 2014), the magnitude of childhood obesity in the United States remains high; approximately 12.5 million (17%) children are classified as obese (Ogden et al. 2012).

High caloric diets and low physical activity levels are accepted as risk factors for obesity; however, the extent of obesity prevalence cannot be entirely explained by these risk factors (Newbold et al. 2009). An emerging hypothesis suggests that environmental exposures may play a role in the onset of childhood obesity (Holtcamp 2012; Thayer et al. 2012); specifically, exposure to secondhand smoke (SHS) may be involved in the onset of childhood obesity. Exposure to SHS is independently associated with increased inflammatory responses, oxidative stress, and endocrine disruption (Barnoya and Glantz 2005; Tziomalos and Charsoulis 2004), and these adverse health effects could ultimately lead to obesity (Tziomalos and Charsoulis 2004; Youn et al. 2014). Furthermore, several epidemiologic studies have reported that self-reported exposure to SHS was positively associated with obesity among children < 10 years of age (Apfelbacher et al. 2008; Kwok et al. 2010; Mangrio et al. 2010; Raum et al. 2011; von Kries et al. 2008; Wen et al. 2013; Yang et al. 2013).

Although the epidemiologic evidence is growing, there remain important gaps in the literature evaluating the impact of exposure to SHS on childhood obesity. Specifically, previous studies may be limited by exposure assessment because self-report of exposure to SHS may not be as accurate as biological markers of exposure (Goniewicz et al. 2011). Cotinine is a nicotine metabolite with a half-life of 16 hr, and 4-(methylnitrosamino)-1-(3-pyridyl)-1-butanol (NNAL) is a tobacco-specific metabolite with a half-life of 10–16 days (Hecht et al. 2001). The use of biomarkers could reduce measurement error; however, to our knowledge, no published studies have evaluated the association between exposure to SHS and childhood obesity using cotinine or NNAL to characterize exposure to SHS.

It is also possible that the joint effect of poor diet and SHS exposures on childhood obesity may be more than would be expected based on the individual effects. Previous epidemiologic evidence indicates that the interaction between active smoking and poor diet (a low Framingham Nutritional Risk Score) on weight gain among adults is more than additive (Kimokoti et al. 2010). An animal study also demonstrated that the association between prenatal exposure to nicotine and subsequent weight gain was stronger among rats exposed to a postnatal high-fat diet (Somm et al. 2008). It is possible that high intakes of fiber, antioxidants, or omega-3 polyunsaturated fatty acids may counteract the inflammatory responses and oxidative stress induced by exposure to SHS (Barnoya and Glantz 2005; Ma et al. 2008; Romieu et al. 2008) and thus reduce the risk for adiposity (Fernández-Sánchez et al. 2011); however, no published studies have explored the potential interactions between exposure to SHS and dietary factors on childhood obesity (Behl et al. 2013).

We evaluated the interaction between exposure to SHS and selected dietary nutrients on the prevalence of obesity among 6- to 19-year-olds using data from the National Health and Nutrition Examination Survey (NHANES) 2007–2010 (CDC 2015). In this analysis, we compared self-reported exposure to SHS with two biomarkers of exposure to SHS (cotinine and NNAL).

## Methods

### Study Population

NHANES is a population-based, cross-sectional survey that uses a complex, multistage approach designed to achieve a nationally representative sample of the U.S. civilian population (CDC 2015). The CDC maintains that institutional review board approval for NHANES, and informed consent was obtained from all participants. Trained interviewers administered surveys in participants’ homes to ascertain information on demographic factors, physical activity, and diet. Children < 16 years of age answered questions with the assistance of an adult household member; children > 16 years of age completed the survey unassisted. An exception was with the administration of the dietary recalls, for which children < 12 years completed the dietary recalls with the assistance of an adult household member and children > 12 years of age completed the dietary recalls without assistance. Additionally, physical examinations and laboratory testing using blood and urine samples were conducted at mobile examination centers.

Urinary NNAL was first measured in NHANES during the 2007–2008 sampling cycle. Therefore, we used NHANES data obtained for 6- to 11-year-olds and 12- to 19-year-olds for the sampling cycles 2007–2008 (*n* = 2,500) and 2009–2010 (*n* = 2,596). We excluded children who were missing body mass index (BMI), laboratory measurements of serum cotinine or urinary NNAL, dietary information, or other physical activity information (*n* = 2,249). We further excluded children with evidence of active smoking, defined as having a cotinine level > 15 ng/mL and/or self-report of current active smoking (*n* = 177, 8%) (Weitzman et al. 2005). Therefore, our final sample size was 2,670.

### Overweight and Obesity

Height was measured using a stadiometer with a fixed vertical backboard and an adjustable headpiece. Weight was measured in kilograms using a digital scale. BMI was calculated for all children by dividing weight (kilograms) by height (meters) squared. Each child’s BMI was converted to an age- and sex-specific *z*-score based on the CDC’s BMI-for-age charts for boys and girls (Kuczmarski et al. 2002). The growth charts were then used to identify the corresponding *z*-scores for overweight (BMI ≥ 85th percentile to BMI < 95th percentile) and obesity (BMI ≥ 95th percentile) (Kuczmarski et al. 2002). Underweight was defined as having a BMI below the 5th percentile, and normal weight was defined as having a BMI at or above the 5th percentile and below the 85th percentile, for age and sex. Because of the small number of underweight children in our sample (*n* = 77; 2.8%), we combined underweight and normal into one category.

As a sensitivity analysis, we also used an international definition of overweight and obesity among children, as defined by the International Obesity Task Force (IOTF) (Cole et al. 2000). The IOTF developed BMI cut-off values for childhood overweight and obesity based on large data sets from six countries including Brazil, Britain, Hong Kong, the Netherlands, Singapore and the United States. These cut-off values are linked with the adult cut-off values of 25 and 30 for overweight and obesity, respectively, by age and sex. In general, there is very strong agreement between the CDC and IOTF definitions in the assessment of the prevalence of overweight/obesity among children (Hajian-Tilaki and Heidari 2013).

### Exposure to Secondhand Smoke

NNAL was measured in spot urine samples using liquid chromatography linked to tandem mass spectrometry (LC/MS/MS). The detection limits have changed over time in NHANES: In 2007–2008, the limit of detection (LOD) was 0.001 ng/mL; in 2009–2010, the LOD was 0.0006 ng/mL. For consistency, we classified all samples with an NNAL level < 0.001 ng/mL as being below the LOD (Clair et al. 2011). The coefficients of variation for NNAL ranged from 5.0% to 10.1% in 2007–2008; the coefficients of variation for 2009–2010 are not available. To account for urinary dilution, standardized concentrations were created by dividing NNAL by urinary creatinine (Avila-Tang et al. 2013). Although there are no established cut-off points for NNAL to classify exposure to SHS, we used methods similar to a previous study evaluating exposure to SHS among nonsmoking adults (Goniewicz et al. 2011). Creatinine-adjusted NNAL was categorized as below the LOD (NNAL < 0.001 ng/mL), low exposure [NNAL ≥ 0.001 ng/mL and ≤ 0.005 ng/mL creatinine (the median value among samples above the LOD)], and high exposure (NNAL > 0.005 ng/mL creatinine). NNAL was the primary indicator of exposure to SHS.

Serum cotinine was measured by isotope dilution–high performance liquid chromatography/atmospheric pressure chemical ionization tandem mass spectrometry (ID HPLC-APCI MS/MS; LOD = 0.015 ng/mL). The coefficients of variation for cotinine ranged from 3.6% to 7.7% among low control batches and 3.3% to 4.8% among high control batches in 2007–2008 and 4.0% to 9.0% among low controls and 3.8% to 5.0% among high controls in 2009–2010. Cotinine was categorized as no exposure using a cut point used by previous studies evaluating a similar hypothesis (cotinine < 0.05 ng/mL) (Clair et al. 2011; Weitzman et al. 2005), low exposure [cotinine ≥ 0.05 ng/mL and ≤ 0.268 ng/mL (the median value among samples above 0.05 ng/mL)] and high exposure (cotinine > 0.268 ng/mL). Self-report of household smokers was categorized as none (no household smokers), low exposure (one household smoker), and high exposure (two or more household smokers).

### Diet

NHANES measured total dietary intake by administering two consecutive 24-hr dietary recalls conducted in person by trained interviewers. The nutrient values for the dietary recalls were based on values in the U.S. Department of Agriculture National Nutrient Database for Standard Reference (USDA 2012). For the present study, we evaluated diet in terms of individual nutrients that we hypothesized might lessen SHS-induced metabolic responses, including dietary fiber, omega-3 polyunsaturated fatty acids [eicosapentaenoic acid (EPA), docosahexaenoic acid (DHA)], vitamin C, and vitamin E. Dietary nutrients were categorized based on the median level.

### Covariates

NHANES collected detailed information about the participant’s household income and family size during the household interview. The poverty index ratio, as calculated by NHANES, is a measure of family income divided by the poverty level determined by the Department of Health and Human Services’ poverty guidelines, specific to family size, year of interview, and state of interview. The poverty index ratio was dichotomized at 1.85, the level used to qualify for federal assistance programs, such as the Women, Infants, and Children (WIC) program (USDA 2015). Among 6- to 11-year-olds, children were asked how many of the past 7 days he or she spent being physically active for at least 60 min (2007–2008) or played or exercised hard enough to sweat for at least 60 min (2009–2010). Among 12- to 19-year-olds, children were asked to identify the number of minutes per day and days per week in the past week they had engaged in moderate activity or vigorous activity. These variables were dichotomized based on the recommendation for children to get at least 60 min of moderate-to-vigorous intensity physical activity every day (Strong et al. 2005). Report of maternal smoking during pregnancy was ascertained by asking the parent/guardian if the biological mother smoked during pregnancy.

### Statistical Methods

All analyses accounted for the complex survey design and NHANES probabilistic sampling weights using the svy commands in Stata version 13 (StataCorp LP). Weighted multinomial logistic regression models were used to describe the interaction between exposure to SHS and dietary variables on the prevalence of overweight and obesity as separate outcomes (compared with normal/underweight). All models adjusted for sex, age (continuous), race/ethnicity (Mexican American, other Hispanic, non-Hispanic white, non-Hispanic black, or other/multiracial), and poverty index ratio (above poverty level or below poverty level) based on previous publications. The ado-command svylogitgof was used to evaluate the F-adjusted mean residual test, a test specifically developed to assess goodness-of-fit for data from a complex survey design (Archer et al. 2007); the test suggested that our final models were a good fit for the data (*p*-value for models > 0.05).

We examined interaction on both the multiplicative scale and the additive scale (Knol and VanderWeele 2012). Interaction by diet was assessed by introducing product terms between dichotomous exposure to SHS (high exposure vs. other) and dichotomized diet variables in separate models. For additive interaction, we used the relative excess risk due to interaction (RERI). The RERI is defined as odds ratio (OR)_11_ – OR_10_ – OR_01_ + 1, where an RERI value of 0 suggests a perfectly additive interaction. We calculated 95% confidence intervals (CIs) and corresponding *p*-values for the RERI values using the method of variance estimates recovery (MOVER) method as described by Zou (2008). For the multiplicative interaction, we calculated *p*-values to assess the statistical significance of the product term.

### Sensitivity Analyses

We conducted several sensitivity analyses. In addition to adjusting for the minimum set of confounders, we additionally adjusted the main effects models for total caloric intake (continuous) and moderate-to-vigorous physical activity (met the recommendations for 60 min/day or did not meet the recommendations) to assess the impact of these potential confounders; these covariates were not included in the main effects analyses due to potential measurement error. Additionally, we adjusted for report of maternal smoking during pregnancy (none or any)—a covariate that is associated with an increased risk of obesity among children exposed to SHS prenatally (Oken et al. 2008) and may explain some of the same variability as exposure to SHS. Because a considerable percentage of children were missing information about maternal smoking during pregnancy (*n* = 654, 24%), this covariate was not included in the main effects analyses. To compare results of models with and without maternal smoking as a covariate, we also limited our main effects analyses to those with information about maternal smoking during pregnancy in a sensitivity analysis (*n* = 2,106). We did not adjust for these covariates in the interaction analyses because the sample size was limited.

We also ran the models using cotinine and by self-report of household smokers to describe exposure to SHS. Additionally, the models were run using the international definition of childhood overweight and obesity. Underweight children (*n* = 77) were also excluded in a sensitivity analysis. Finally, we investigated age groups separately (ages 6–11 years and ages 12–19 years) and the survey cycles separately (2007–2008 and 2009–2010).

## Results

Weighted proportions of weight status and exposure to SHS are shown in Table 1. One third of children were either overweight (15%) or obese (19%). Approximately half of the children had levels of creatinine-adjusted NNAL and cotinine below the limit of detection (53% and 57%, respectively), and most children (87%) reported no smokers within the household.

**Table 1:** Weighted proportions of weight status and exposure to SHS among 6- to 19-year-olds, 2007–2010 NHANES (*n* = 2,670).

Characteristic	Percent (95% CI)
Weight categories
U.S. definition^*a*^
Normal/underweight	66 (64, 68)
Overweight	15 (14, 16)
Obese	19 (17, 21)
International definition^*b*^
Normal/underweight	65 (63, 67)
Overweight	20 (19, 22)
Obese	15 (13, 16)
Exposure assessment
NNAL exposure
Below LOD (< 0.001 ng/mL creatinine)	53 (48, 57)
Low (≥ 0.001 & < 0.005 ng/mL creatinine)	24 (21, 27)
High (≥ 0.005 & < 0.082 ng/mL creatinine)	23 (18, 25)
Cotinine exposure
No (< 0.05 ng/mL)	57 (53, 61)
Low (≥ 0.05 & < 0.0268 ng/mL)	21 (19, 24)
High (≥ 0.268 & < 14.6 ng/mL)	22 (18, 25)
Self-report of household smokers
None	86 (85, 89)
One	8 (7, 10)
Two or more	6 (4, 9)
Abbreviations: CI, confidence intervals; LOD, limit of detection; NHANES, National Health and Nutrition Examination Survey; NNAL, 4-(methylnitrosamino)-1-(3-pyridyl)-1-butanol; SHS, secondhand smoke. The total estimated population using the sampling weights is 31,119,675. ^***a***^Overweight was defined as having a body mass index ≥ 85th percentile and < 95th percentile and obesity was defined as having a body mass index ≥ 95th percentile by age and sex, based on the 2000 CDC growth charts. ^***b***^Overweight and obesity is defined as having a body mass index that corresponds to a body mass index of 25 and 30 at age 18 years, respectively, based on the International Obesity Task Force growth charts.	

Table 2 presents weighted proportions of exposure to SHS and covariates by weight status categories. Exposure status was slightly different across the weight status categories. The proportion of children who had high creatinine-adjusted NNAL levels was 21% among children who were classified as normal/underweight, 23% among children who were classified as overweight, and 32% among children who were classified as obese. The mean age was 12 years across the weight status categories, and a greater proportion of males than females were classified as obese (56% and 44%, respectively). Race/ethnic proportions were slightly different across the weight status categories; for instance, the proportion of non-Hispanic white children was 62% among those classified as normal/underweight, 53% among those classified as overweight, and 51% among those classified as obese. The proportion of children who were below the poverty level was higher among children who were classified as obese than among children who were classified as normal/underweight. In general, a majority of the children reported that they met the recommendations for children to get at least 60 min of moderate-to-vigorous intensity physical activity every day. The distributions of weight status, exposure to SHS, and covariates for the separate age groups (6- to 11-year-olds and 12- to 19-year-olds) and survey cycles (2007–2008 and 2009–2010) were similar to the findings for age groups and survey cycles combined (results not presented). Compared with children with information about report of maternal smoking during pregnancy (*n* = 2,106), children who were missing information (*n* = 654) were more likely to be female, to be white, to have a poverty index ratio above the poverty level, and to have high NNAL levels (results not presented).

**Table 2:** Weighted proportions by weight status*^a^* of U.S. children, ages 6–19 years, 2007–2010 NHANES, *n* = 2,670.

Variable	Percent (95% CI)		
	Normal/underweight	Overweight	Obese
Exposure assessment
NNAL
Below LOD (< 0.001 ng/mL creatinine)	57 (52, 62)	49 (46, 54)	39 (33, 47)
Low (≥ 0.001 and < 0.005 ng/mL creatinine)	22 (18, 26)	28 (25, 31)	28 (28, 34)
High (≥ 0.005 and < 0.082 ng/mL creatinine)	21 (18, 25)	23 (19, 25)	32 (26, 40)
Cotinine
No (< 0.05 ng/mL)	60 (56, 64)	57 (53, 61)	47 (40, 54)
Low (≥ 0.05 and < 0.268 ng/mL)	21 (18, 24)	23 (20, 25)	22 (17, 27)
High (≥ 0.268 and < 14.6 ng/mL)	19 (16, 22)	19 (15, 23)	31 (25, 29)
Self-report of household smokers
None	88 (85, 90)	89 (87, 92)	78 (71, 83)
One	6 (5, 8)	8 (7, 10)	12 (8, 17)
Two or more	6 (4, 9)	4 (3, 8)	10 (6, 17)
Covariates
Age (years, mean)	12.3 (12.0, 12.6)	12.5 (12.2, 12.8)	12.4 (12.0, 12.7)
Sex
Male	51 (47, 54)	52 (50, 54)	56 (51, 61)
Female	49 (46, 52)	48 (46, 51)	44 (39, 49)
Race/ethnicity
Non-Hispanic white	62 (56, 67)	53 (47, 59)	51 (41, 60)
Non-Hispanic black	13 (10, 15)	17 (11, 25)	19 (13, 28)
Mexican American	12 (9, 16)	17 (11, 19)	17 (13, 21)
Other Hispanic	8 (5, 10)	6 (4, 9)	5 (4, 6)
Other/multiracial	6 (4, 9)	6 (5, 9)	5 (3, 7)
Poverty index ratio^*b*^
Above poverty level (≥ 1.85)	62 (57, 67)	58 (54, 65)	51 (44, 58)
Below poverty level (< 1.85)	38 (33, 43)	42 (37, 47)	49 (41, 55)
Moderate-to-vigorous physical activity
Met recommendations for 60 min/day	87 (82, 90)	84 (81, 87)	86 (83, 89)
Did not meet recommendations	13 (10, 17)	16 (13, 19)	14 (11, 17)
Report of maternal smoking during pregnancy^*c*^
None	87 (84, 91)	85 (78, 89)	82 (76, 86)
Any	12 (9, 16)	15 (11, 21)	18 (14, 24)
Abbreviations: CI, confidence intervals; LOD, limit of detection; NHANES, National Health and Nutrition Examination Survey; NNAL, 4-(methylnitrosamino)-1-(3-pyridyl)-1-butanol. The total estimated population using the sampling weights is 31,119,675. ^***a***^Overweight was defined as having a body mass index ≥ 85th percentile and < 95th percentile, and obesity was defined as having a body mass index ≥ 95th percentile by age and sex, based on the 2000 CDC growth charts. ^***b***^The poverty index ratio was dichotomized at 1.85, the level used to qualify for federal assistance programs, such as the Women, Infants, and Children program. ^***c***^Estimates for report of maternal smoking during pregnancy are based on a different sample size due to missing information (*n* = 2,106).			

### Exposure to Secondhand Smoke

Among those who reported no smokers in the household, 41% had a creatinine-adjusted NNAL level above the LOD and 35% had a cotinine level above the LOD (Table 3). Among children with high levels of NNAL or cotinine, approximately one-third also reported any maternal smoking during pregnancy (33% and 35%, respectively); however, nearly half (45%) of the children who reported living with two or more household smokers reported any maternal smoking during pregnancy.

**Table 3:** Comparison of exposure to SHS categories among 6- to 19-year-olds, 2007–2010 NHANES (%).

Exposure to SHS	Maternal report of smoking during pregnancy^*a*^^,^^*b*^		NNAL^*c*^			Cotinine		
	None	Any	Below LOD	Low	High	No	Low	High
NNAL
Below LOD	96	4	—	—	—	—	—	—
Low	90	10	—	—	—	—	—	—
High	67	33	—	—	—	—	—	—
Cotinine
No	97	3	78	20	2	—	—	—
Low	86	14	29	50	21	—	—	—
High	65	35	4	10	86	—	—	—
Self-report of household smokers
None	93	7	59	27	14	65	24	11
One	59	41	6	13	81	4	16	81
Two or more	55	45	2	9	89	4	7	89
Abbreviations: LOD, limit of detection; NHANES; National Health and Nutrition Examination Survey; NNAL, 4-(methylnitrosamino)-1-(3-pyridyl)-1-butanol; SHS, secondhand smoke. ^***a***^Serum cotinine categories were as follows: no (< 0.05 ng/mL), low (≥ 0.05 and < 0.268 ng/mL), and high (≥ 0.268 and < 14.6 ng/mL).^***b***^Estimates for report of maternal smoking during pregnancy are based on a different sample size due to missing information (*n* = 2,106). ^***c***^Urinary NNAL categories were as follows: below LOD (< 0.001 ng/mL creatinine), low (≥ 0.001 and < 0.005 ng/mL creatinine), and high (≥ 0.005 and < 0.082 ng/mL creatinine).								

### Overweight and Obesity

The proportions of children who were classified as underweight/normal using the U.S. and international definitions were similar (Table 1). There was some variation in how the U.S. definition and the international definition classified overweight and obesity. Specifically, among children who were classified as overweight using the international definition, approximately 24% were classified as normal/underweight using the U.S. definition (see Table S1). An overwhelming majority of the children (98%) who were classified as obese using the international definition were also classified as obese using the U.S. definition.

### Diet

The correlations between dietary fiber, vitamin C, vitamin E, DHA, and EPA are shown in Table S2. There was a moderate correlation between DHA and EPA (Spearman’s rank correlation coefficient = 0.70) and between dietary fiber and vitamin E (Spearman’s rank correlation coefficient = 0.65). However, the remaining dietary nutrients were weakly correlated (Spearman’s rank correlation coefficients ranging from 0.08 to 0.39).

### Interaction Analysis

The additive and multiplicative interaction results suggested that the prevalence of obesity among children with both high NNAL levels and low levels of certain nutrients (dietary fiber, DHA, or EPA) were greater than would be expected due to the estimated effects of the individual exposures alone (Table 4). For example, children with high NNAL levels and low fiber intakes were more than twice as likely to be obese than were children with low NNAL levels and high fiber intakes. The RERI was 0.8 (95% CI: 0.1, 1.5), which indicates that the joint effect of high NNAL levels and low fiber intakes is higher than expected based on the sum of the individual effects (observed OR = 2.6; 95% CI: 1.6, 4.0, and expected OR = 1.8; 1.7 + 1.1–1.0). The stratified results indicate that the effect of high NNAL levels on obesity prevalence was stronger among children with low fiber intakes (OR = 2.4; 95% CI: 1.7, 3.3) than among children with high fiber intakes (OR = 1.7; 95% CI: 1.2, 2.3). The estimated joint effects of high NNAL and low dietary intakes on overweight did not indicate differences from additive or multiplicative effects of either exposure alone. The association between SHS and obesity did not appear to be modified by dietary vitamins C or E.

**Table 4:** Adjusted*^a^* ORs and 95% CIs for overweight and obesity*^b^* in relation to exposure to SHS and dietary nutrients and measures of additive*^c^* and multiplicative*^d^* interaction among 6- to 19-year-olds, 2007–2010 NHANES.

Dietary nutrient	NNAL exposure	Overweight vs. normal/underweight		Obese vs. normal/underweight	
		OR (95% CI)	Stratified OR (95% CI)	OR (95% CI)	Stratified OR (95% CI)
Fiber
High fiber intake (≥ 12.75 g/day)	Below LOD/low	1^*e*^	1	1	1
	High	1.1 (0.8, 1.6)	1.1 (0.8, 1.6)	1.7 (1.2, 2.3)	1.7 (1.2, 2.3)
Low fiber intake (< 12.75 g/day)	Below LOD/low	1.1 (0.7, 1.5)	1	1.1 (0.8, 1.4)	1
	High	1.6 (1.0, 2.6)	1.5 (0.9, 2.3)	2.6 (1.6, 4.0)	2.4 (1.7, 3.3)
*p* for multiplicative interaction		*p* = 0.47		*p* = 0.05
RERI (95% CI); *p* for additive interaction		0.4 (–0.2, 1.0); *p* = 0.19		0.8 (0.1, 1.5); *p* = 0.03
EPA
High EPA intake (≥ 0.007 g/day)	Below LOD/low	1	1	1	1
	High	1.4 (0.9, 2.0)	1.4 (0.9, 2.0)	1.6 (1.1, 2.3)	1.6 (1.1, 2.3)
Low EPA intake (< 0.007 g/day)	Below LOD/low	1.2 (0.8, 1.8)	1	1.0 (0.8, 1.3)	1
	High	1.4 (0.9, 2.3)	1.2 (0.7, 2.2)	2.6 (2.0, 3.5)	2.6 (1.9, 4.0)
*p* for multiplicative interaction		*p* = 0.76		*p* = 0.05
RERI (95% CI); *p* for additive interaction		–0.2 (–0.9, 0.5); *p* = 0.56		1.0 (0.3, 1.8); *p* = 0.01
DHA
High DHA intake (≥ 0.018 g/day)	Below LOD/low	1	1	1	1
	High	1.2 (0.8, 1.9)	1.2 (0.8, 1.9)	1.6 (1.0, 2.5)	1.6 (1.0, 2.5)
Low DHA intake (< 0.018 g/day)	Below LOD/low	1.2 (0.9, 1.7)	1	1.0 (0.8, 1.4)	1
	High	1.7 (0.9, 2.7)	1.4 (0.8, 2.4)	2.4 (1.7, 3.4)	2.4 (1.6, 3.5)
*p* for multiplicative interaction		*p* = 0.68		*p* = 0.19
RERI (95% CI); *p* for additive interaction		0.3 (–0.4, 1.0); *p* = 0.41		0.8 (0.1, 1.6); *p* = 0.04
Vitamin C
High vitamin C intake (≥ 68.9 g/day)	Below LOD/low	1	1	1	1
	High	1.3 (0.9, 1.9)	1.3 (0.9, 1.9)	1.8 (1.3, 2.6)	1.8 (1.3, 2.6)
Low vitamin C intake (< 68.9 g/day)	Below LOD/low	1.2 (0.8, 1.8)	1	1.1 (0.8, 1.5)	1
	High	1.7 (1.0, 2.7)	1.4 (0.7, 2.4)	2.4 (1.7, 3.4)	2.2 (1.5, 3.6)
*p* for multiplicative interaction		*p* = 0.78		*p* = 0.30
RERI (95% CI); *p* for additive interaction		0.2 (–0.4, 0.9); *p* = 0.56		0.5 (–0.2, 1.3); *p* = 0.18
Vitamin E
High vitamin E intake (≥ 5.415 mg/day)	Below LOD/low	1	1	1	1
	High	1.2 (0.9, 1.7)	1.2 (0.9, 1.7)	1.9 (1.4, 2.6)	1.9 (1.4, 2.6)
Low vitamin E intake (< 5.415 mg/day)	Below LOD/low	1.5 (1.1, 2.0)	1	1.2 (0.9, 1.5)	1
	High	2.2 (1.5, 3.3)	1.5 (0.9, 2.3)	2.6 (1.8, 3.7)	2.2 (1.5, 3.2)
*p* for multiplicative interaction		*p* = 0.34		*p* = 0.56
RERI (95% CI); *p* for additive interaction		0.5 (–0.2, 1.3); *p* = 0.20		0.5 (–0.3, 1.3); *p* = 0.22
Abbreviations: CI, confidence intervals; DHA, docosahexaenoic acid; EPA, eicosapentaenoic acid; LOD, limit of detection; NHANES; National Health and Nutrition Examination Survey; NNAL, 4-(methylnitrosamino)-1-(3-pyridyl)-1-butanol; OR, odds ratio; RERI, relative excessive risk due to interaction; SHS, secondhand smoke. ^***a***^Adjusted for sex, age, race/ethnicity, and poverty index ratio. ^***b***^Overweight was defined as having a body mass index ≥ 85th percentile and < 95th percentile and obesity was defined as having a body mass index ≥ 95th percentile by age and sex, based on the 2000 CDC growth charts. ^***c***^*p* for additive interaction generated for the relative excess risk due to interaction value. ^***d***^*p* for multiplicative interaction generated for the product term of each dietary factor (e.g., fiber, EPA, DHA) and exposure to SHS. ^***e***^Reference category.					

### Sensitivity Analyses

There was a positive association between exposure to SHS and obesity; children with high NNAL levels were more than twice as likely to be obese than were children with low NNAL levels, after adjusting for sex, age, race/ethnicity, and poverty index ratio (OR = 2.6; 95% CI: 1.6, 3.1). The association between exposure to SHS and obesity was not changed following adjustment for total caloric intake and physical activity levels; however, the association was slightly attenuated following adjustment for report of maternal smoking during pregnancy (see Table S3). The main effects and interaction results were consistent when exposure to SHS was determined by cotinine and by self-report of household smokers and when we used the international definition of childhood overweight and obesity (results not shown). After excluding underweight children from our analyses (*n* = 77), we found no meaningful impact on the main effects results (results not presented). Furthermore, the main effects results for the separate age groups and survey cycles were similar to the results for age groups and survey cycles combined (results not shown).

## Discussion

The results of this study suggest that the joint effects of high exposure to SHS and low levels of certain nutrients (dietary fiber, DHA, or EPA) on obesity were greater than would be expected due to the effects of the individual exposures alone. For example, children with high NNAL levels and low fiber intakes were more than twice as likely to be obese than were children with low NNAL levels and high fiber intakes. Furthermore, the associations between exposure to SHS and obesity were stronger among children with low intakes of dietary fiber, EPA, and DHA compared with children with high intakes of these nutrients. Our results are consistent with a number of previous studies evaluating the independent associations between exposure to SHS and childhood obesity, and our identification of statistical interaction with various dietary factors may support the hypothesized biological mechanisms of these associations.

Many compounds found in SHS, including nicotine and polycyclic aromatic hydrocarbons, are suspected endocrine disruptors and could negatively affect the utilization of insulin and promote metabolic imbalance (Tziomalos and Charsoulis 2004). Other potential pathways linking SHS exposures to obesity have been hypothesized; exposure to SHS is independently associated with inflammation and systemic oxidative stress (Barnoya and Glantz 2005), which could play a role in the development of obesity (Youn et al. 2014).

The inflammatory responses, oxidative stress, and endocrine disruption responses due to SHS may be counteracted by high intakes of dietary fiber and omega-3 polyunsaturated fatty acids. High dietary fiber may reduce the harmful effects of SHS exposures by reducing inflammatory responses (Ma et al. 2008). Previous research has indicated that high dietary fiber consumption may ameliorate the harmful effects of exposure to SHS on the risk of coronary heart disease mortality among adults (Clark et al. 2013). Omega-3 polyunsaturated fatty acids may also modulate the adverse effects of environmental exposures by reducing the generation of reactive oxygen species (Romieu et al. 2008). Additionally, one *in vitro* study demonstrated that high intakes of EPA may also inhibit apoptosis caused by nicotine-derived nitrosamino ketone (NNK), the precursor to NNAL (Tithof et al. 2001). These potential mechanisms are supported by two prospective cohort studies which observed that omega-3 polyunsaturated fatty acids modified the association between smoking and coronary heart disease incidence, one among 8,006 Japanese-American men 45–65 years of age who lived in Hawaii (Rodriguez et al. 1996) and one among 72,012 Japanese men and women 45–74 years of age (Eshak et al. 2014).

Previous studies have consistently observed positive associations between exposure to SHS and childhood obesity. One prospective cohort study of 21,083 mother–child pairs in the U.S. Collaborative Perinatal Project evaluated the association between exposure to SHS and childhood obesity; Wen et al. (2013) observed that heavy maternal smoking (≥ 20 cigarettes/day) was associated with obesity among children at 7 years of age compared with no maternal smoking (adjusted OR = 1.49; 95% CI: 1.31, 1.69). These findings are supported by published observational studies. For instance, Raum et al. (2011) observed that children whose parents reported child’s exposure to SHS at age 1 year and at age 6 years had higher odds for obesity (adjusted OR = 2.90; 95% CI: 1.86, 4.54) compared with children whose parents reported no exposure to SHS. The largest study, conducted among a sample of 35,434 children ages 5–7 years, observed that parental self-report of household smoking was associated with childhood obesity (adjusted OR = 1.13; 95% CI: 0.98, 1.32) (Apfelbacher et al. 2008).

Strong evidence already exists for the increased risk of obesity among children exposed to SHS prenatally; a recent meta-analysis estimated that maternal smoking during pregnancy increases the risk for obesity among children by 50% (Oken et al. 2008). To distinguish the effects of prenatal and postnatal exposure to SHS on childhood obesity (Behl et al. 2013), we adjusted for report of maternal smoking during pregnancy in sensitivity analyses. We observed a slight attenuation in the association between exposure to SHS and obesity in the main effects models following adjustment for report of maternal smoking during pregnancy (see Table S3). For example, the OR for high NNAL levels before adjustment was 2.5 (95% CI: 1.7, 3.5) and the OR for high NNAL levels after adjusting for maternal smoking during pregnancy was 1.8 (95% CI: 1.2, 2.7). Because a large portion of children was missing information about maternal smoking during pregnancy, we also limited our analyses to those with information about maternal smoking during pregnancy (*n* = 2,106) and observed only a slight decrease in the odds for childhood obesity. For example, the OR for high NNAL levels among the restricted population (OR = 2.1; 95% CI: 1.4, 3.2) was similar to the OR for high NNAL levels following adjustment for maternal smoking during pregnancy (OR = 1.8; 95% CI: 1.2, 2.7).

This study provides insight about the utility of three different exposure metrics for evaluating the impact of exposure to SHS on childhood obesity. Contrary to what was expected, our results suggest that the association between exposure to SHS and obesity were consistent regardless of whether SHS was characterized by self-report, cotinine, or NNAL. Self-report of household smokers was limited to exposures within the home and did not attempt to capture exposure in other settings (e.g., schools, workplaces for older children, other households, multiunit housing), whereas cotinine likely captures the cumulative exposure to SHS over a shorter period of time than NNAL (half-life of 16 hr vs. 10–16 days, respectively) (Avila-Tang et al. 2013). Despite the differences in exposure classification across the three exposure metrics, the associations between SHS exposures and obesity were only slightly stronger for NNAL than for cotinine and self-report of household smokers. Our results suggest that self-report of household smokers or cotinine may be just as appropriate to assess exposure to SHS among children who may be more likely to be exposed while at home. Because self-report and cotinine are easier and less expensive to measure than NNAL (Avila-Tang et al. 2013), one could argue that the latter is not necessary for studies evaluating this particular research question.

Several limitations should be considered when interpreting these results. It is possible that the associations observed in this study are attributable to residual confounding of physical activity and diet, because these covariates are difficult to accurately measure (Thompson et al. 2010). Self-reported physical activity is subject to over-reporting due to social desirability (Prince et al. 2008) and is weakly correlated (*r* < 0.30) with accelerometer-based estimates of physical activity levels (Tucker et al. 2011); these considerations could explain the relatively high proportion of children who met the recommendations for physical activity. There may be some limitations in how physical activity was measured as well. On the other hand, NHANES performs two consecutive 24-hr dietary recalls to evaluate diet, which may have eliminated some of the issues of a single measurement. Additionally, we evaluated confounding by diet in terms of total caloric intake, and there was no meaningful impact on the results following adjustment for the nutrient patterns. Our results may also be affected by our inability to adjust for other important covariates, such as parental BMI, because these variables were not available in the NHANES data set.

Although the temporality of the relationship between exposure to SHS and obesity cannot be established, this study is a useful first step toward evaluating these interaction associations and provides evidence supporting the need for future investigation in larger-scale, prospective analyses. An important strength of the present study is the sampling methods and the complex survey design employed by NHANES, which allows for the results to be generalized to all U.S. children.

## Conclusion

Low levels of dietary fiber and omega-3 polyunsaturated fatty acids may worsen the effects of exposure to SHS on childhood obesity. Childhood obesity prevention strategies aimed at reducing SHS exposures and improving diets may exceed the expected benefits based on targeting either risk factor alone.

## Supplemental Material

(198 KB) PDF
