# Supplementary material for: Interactions between Diet and Exposure to Secondhand Smoke on the Prevalence of Childhood Obesity: Results from NHANES, 2007–2010
Source: Environ Health Perspect. 2015 Dec 29;124(8):1316–22. doi: 10.1289/ehp.1510138 (PMC4977048; doi:10.1289/ehp.1510138)
Supplement: (198 KB) PDF [file ehp.1510138.s001.acco.pdf]

Environ Health Perspect
DOI: 10.1289/ehp.1510138

Note to readers with disabilities: *EHP* strives to ensure that all journal content is accessible to all readers. However, some figures and Supplemental Material published in *EHP* articles may not conform to 508 standards due to the complexity of the information being presented. If you need assistance accessing journal content, please contact ehp508@niehs.nih.gov. Our staff will work with you to assess and meet your accessibility needs within 3 working days.

Supplemental Material

Interactions between Diet and Exposure to Secondhand Smoke on the Prevalence of Childhood Obesity: Results from NHANES, 2007–2010

Brianna F. Moore, Maggie L. Clark, Annette Bachand, Stephen J. Reynolds, Tracy L. Nelson, and Jennifer L. Peel

Table of Contents

Table S1. Comparison of weight categories among 6-19 year olds, 2007-2010 NHANES

Table S2. Spearman rank correlation coefficients for dietary nutrients among 6-19 year olds, 2007-2010 NHANES

Table S3. Crude and adjusted models for the association of exposure to SHS exposure and overweight and obesity[a] among U.S. children, ages 6-19 years, 2007-2010 NHANES

Table S1. Comparison of weight categories among 6-19 year olds, 2007-2010 NHANES

	**U.S. Definition**[a]		
	Normal/underweight	Overweight	Obese
**International Definition**[b]			
Normal/underweight	99%	1%	0%
Overweight	24%	68%	8%
Obese	0%	2%	98%

Abbreviations: NHANES, National Health and Nutrition Examination Survey

[a]Overweight was defined as having a body mass index ≥85th percentile and <95th percentile and obesity was defined as having a body mass index ≥95th percentile by age and sex, based on the 2000 Centers for Disease Control and Prevention growth charts.

[b]Overweight and obesity is defined as having a body mass index that corresponds to a body mass index of 25 and 30 at age 18, respectively, based on the International Obesity Task Force growth charts.

DOI: 10.1289/ehp.1510138

Table S2. Spearman rank correlation coefficients for dietary nutrients among 6-19 year olds, 2007-2010 NHANES

	Dietary Fiber	Vitamin C	Vitamin E	EPA	DHA
Dietary Fiber	1				
Vitamin C	0.39	1			
Vitamin E	0.65	0.36	1		
EPA	0.18	0.11	0.28	1	
DHA	0.08	0.13	0.22	0.70	1

Abbreviations: DHA, docosahexaenoic acid; EPA, eicosapentaenoic acid; NHANES, National Health and Nutrition Examination Survey

DOI: 10.1289/ehp.1510138

Table S3. Crude and adjusted models for the association of exposure to SHS exposure and overweight and obesity[a] among U.S. children, ages 6-19 years, 2007-2010 NHANES

	**NNAL Exposure**		**Cotinine Exposure**		**Self-report of Household Smokers**	
	**Overweight vs. Normal ORs (95% CIs)**	**Obese vs. Normal ORs (95% CIs)**	**Overweight vs. Normal ORs (95% CIs)**	**Obese vs. Normal ORs (95% CIs)**	**Overweight vs. Normal ORs (95% CIs)**	**Obese vs. Normal ORs (95% CIs)**
**Crude**						
Below LOD/None	1[b]	1	1	1	1	1
Low	1.2 (0.9, 1.6)	1.6 (1.2, 2.3)	1.2 (0.9, 1.6)	1.2 (1.0, 1.5)	1.2 (0.8, 1.9)	1.8 (1.3, 2.3)
High	1.2 (1.0, 1.6)	1.9 (1.4, 2.6)	1.1 (0.1, 1.4)	1.8 (1.4, 2.3)	0.8 (0.4, 1.3)	1.6 (1.0, 2.4)
p for trend[c]	p=0.18	p<0.01	p=0.40	p<0.01	p=0.59	p<0.01
**Model 1[d]**						
Below LOD/None	1	1	1	1	1	1
Low	1.3 (0.9, 1.8)	1.7 (1.2, 2.5)	1.2 (0.9, 1.6)	1.3 (1.0, 1.6)	1.2 (0.7, 1.9)	1.7 (1.2, 2.4)
High	1.3 (1.0, 1.7)	2.2 (1.6, 3.1)	1.2 (0.8, 1.6)	1.9 (1.4, 2.5)	0.8 (0.5, 1.6)	1.7 (1.1, 2.8)
p for trend	p=0.08	p<0.01	p=0.29	p<0.01	p=0.84	p<0.01
**Model 2[e]**						
Below LOD/None	1	1	1	1	1	1
Low	1.5 (1.1, 2.1)	1.8 (1.3, 2.7)	1.3 (0.9, 1.9)	1.6 (1.1, 2.0)	1.2(0.6, 2.4)	2.0 (1.4, 3.0)
High	1.4 (0.9, 2.0)	2.5 (1.7, 3.5)	1.1 (0.8, 1.6)	2.1 (1.5, 3.0)	0.8 (0.3, 1.7)	2.1 (1.3, 3.3)
p for trend	p=0.04	p<0.01	p=0.36	p<0.01	p<0.05	p=0.74
**Model 2b[f]**						
Below LOD/None	1	1	1	1	1	1
Low	1.4 (1.0, 2.1)	2.2 (1.5, 3.2)	1.7 (1.1, 2.6)	1.7 (1.2, 2.7)	1.0 (0.5, 1.9)	1.5 (0.9, 2.5)
High	1.2 (0.8, 1.8)	2.1 (1.4, 3.2)	1.1 (0.7, 1.6)	1.9 (1.3, 2.9)	0.7 (0.3, 1.6)	1.6 (1.0, 2.7)
p for trend	p=0.18	p<0.01	p=0.33	p<0.01	p=0.30	p=0.02
**Model 3[g]**						
Below LOD/None						
Low	1.4 (0.9, 2.1)	2.1 (1.5, 3.0)	0.9 (0.6, 1.4)	1.7 (1.1, 2.5)	0.8 (0.4, 1.7)	1.0 (1.0, 1.1)
High	1.1 (0.7, 1.6)	1.8 (1.2, 2.7)	1.6 (1.1, 2.5)	1.6 (1.1, 2.5)	0.5 (0.2, 1.4)	2.0 (1.4, 2.8)
p for trend	p=0.44	p<0.01	p=0.67	p=0.01	p=0.10	p=0.27

DOI: 10.1289/ehp.1510138

Abbreviations: CI, confidence intervals; LOD, limit of detection; NNAL, 4-(methylnitrosamino)-1-(3-pyridyl)-1-butanol; OR, odds ratio; SHS, secondhand smoke

[a]Overweight was defined as having a body mass index ≥85th percentile and <95th percentile and obesity was defined as having a body mass index ≥95th percentile by age and sex, based on the 2000 Centers for Disease Control and Prevention growth charts.

[b]Reference category.

[c]Tests for trends were performed by treating the categorical exposure to SHS variable as a continuous variable in the logistic regression model.

[c]Adjusted for sex, age, race/ethnicity and poverty index ratio.

[d]Model 1 plus additional adjustment for the total caloric intake and physical activity levels.

[e]Model 1 plus adjustment for the total caloric intake and physical activity levels among children with information about maternal smoking during pregnancy (n=2,106).

[f]Model 2 plus additional adjustment for report of maternal smoking during pregnancy (n=2,106).
